# The Modulatory Effects of *Bacteroides thetaiotaomicron* on Metabolic Parameters, Expression of Diabetes- and Inflammation-Related Genes and Gut Microbiota Composition in a Male Rat Model of Type 2 Diabetes Mellitus

**DOI:** 10.5812/ijem-168629

**Published:** 2025-10-31

**Authors:** Farzaneh Hasanian-Langroudi, Mehdi Hedayati, Asghar Ghasemi, Seyed Davar Siadat, Maryam Tohidi

**Affiliations:** 1 Prevention of Metabolic Disorders Research Center, Research Institute for Metabolic and Obesity Disorders, Research Institute for Endocrine Sciences, Shahid Beheshti University of Medical Sciences, Tehran, Iran; 2 Cellular and Molecular Endocrine Research Center, Research Institute for Endocrine Molecular Biology, Research Institute for Endocrine Sciences, Shahid Beheshti University of Medical Sciences, Tehran, Iran; 3 Endocrine Physiology Research Center, Research Institute for Endocrine Molecular Biology, Research Institute for Endocrine Sciences, Shahid Beheshti University of Medical Sciences, Tehran, Iran; 4 Department of Mycobacteriology and Pulmonary Research, Microbiology Research Center, Pasteur Institute of Iran, Tehran, Iran; 5 Prevention of Metabolic Disorders Research Center, Research Institute for Endocrine Sciences, Shahid Beheshti University of Medical Sciences, Tehran, Iran

**Keywords:** *Bacteroides thetaiotaomicron*, Endocannabinoid Receptors, Gut Microbiota, Rat Model, Type 2 Diabetes Mellitus

## Abstract

**Background:**

Type 2 diabetes mellitus (T2DM) is a prevalent disorder with significant complications and mortality. Gut microbiota plays a role in metabolic homeostasis, and dysbiosis may contribute to inflammation and insulin resistance (IR).

**Objectives:**

This study aimed to investigate the effect of *Bacteroides thetaiotaomicron* on glycemic and IR markers, lipid profiles, and the expression of diabetes- and inflammation-related genes, as well as the abundance of targeted gut microbiota in a rat model of T2DM.

**Methods:**

Thirty-two male Wistar rats were randomly assigned to normal control groups or a high-fat diet/streptozotocin-induced T2DM group. Each group received 5-week oral *B. thetaiotaomicron* (1×10^9^ CFU/mL) or phosphate-buffered saline (PBS). Anthropometric and metabolic measures were compared pre- and post-intervention. Expression of diabetes-related genes (PI3K, Akt) in the liver, inflammation-related genes (IL-6, IL-10, IL-1β, IL-4) in the colon, cannabinoid receptors (CB1, CB2) in both tissues, and changes in gut microbiota composition were evaluated using quantitative PCR.

**Results:**

Compared to the T2DM-PBS group, administration of *B. thetaiotaomicron* to T2DM rats led to significant reductions in body weight (BW) (8%), Body Mass Index (BMI) (21%), Lee index (10%), fasting blood glucose (FBG) (16%), insulin (46%), homeostatic model assessment of IR (HOMA-IR) (56%), triglycerides (TG) (34%), total cholesterol (TC) (29%), and low-density lipoprotein cholesterol (25%) (all P ≤ 0.012). This intervention was associated with reduced expression of CB1 (1.81-fold) and increased expression of PI3K (4.91-fold), Akt (3.55-fold), and CB2 (2.26-fold) (all P < 0.0001). Furthermore, expression of IL-1β (1.76-fold), IL-6 (2.10-fold), and CB1 (1.64-fold) was significantly down-regulated, whereas expression of other inflammation-related genes including IL-4 (2.43-fold), CB2 (1.47-fold), and IL-10 (4.6-fold) was up-regulated (all P ≤ 0.0009). Moreover, significant changes in targeted gut microbiota were observed (a reduction in the abundance of Bacillota and Actinomycetota and an increase in Bacteroidota, *Faecalibacterium prausnitzii*, *B. thetaiotaomicron*, and *Clostridium* cluster IV).

**Conclusions:**

*Bacteroides thetaiotaomicron* improved anthropometric measures, glycemic indices, IR, lipid profiles, and regulated the expression of diabetes- and inflammation-related genes, along with modification of gut microbiota composition in a T2DM rat model.

## 1. Background

Type 2 diabetes mellitus (T2DM) is now regarded as a leading chronic metabolic disease across the world, affecting about 589 million individuals, with forecasts suggesting that adult prevalence will rise to 783 million by 2045 (1). Type 2 diabetes mellitus is an independent and well-established risk factor for various life-threatening conditions and increased mortality (2). Genetic predisposition, obesity, lifestyle, immune disorders, infection, long-term use of antibiotics, and profound alteration in the gut microbiota (dysbiosis) are risk factors for T2DM (3). Male rodents are more prone to obesity, insulin resistance (IR), and hyperglycemia in most animal models, which has led to their predominant use in diabetes research (4). The microbiota refers to a collection of living microorganisms in a specific environment, encompassing bacteria, archaea, and fungi. Gut microbiota influence health in part by producing numerous metabolites from the diet, boosting anti-inflammatory cytokines and chemokines, or inducing low-grade inflammation. Recent studies indicate that dysbiosis may contribute to metabolic inflammation and IR (5). According to previous studies, the predominant forms of gut microbiota that might contribute to T2DM belong to four phyla: Bacillota, Bacteroidota, Pseudomonadota, and Actinomycetota, alongside bacterial genera/species, including *Akkermansia muciniphila* (A. muciniphila), *Clostridium* cluster IV, *Faecalibacterium prausnitzii*, *Lactobacillus* spp., and *Bacteroides thetaiotaomicron* (6, 7). Among these, *B. thetaiotaomicron*, an anaerobe in the intestinal microflora of humans and mice, has attracted attention due to its potential to modulate metabolic pathways, influence glucose metabolism, and regulate the expression of inflammation-related genes (8, 9). *Bacteroides thetaiotaomicron* helps to protect intestinal cells from inflammation by activating the phosphoinositide 3-kinase/protein kinase B (PI3K/Akt) pathway (10). The PI3K/Akt signaling pathway plays a crucial role in IR through modulation of fundamental cellular functions such as proliferation and glucose uptake. This pathway is closely associated with the development of T2DM in the context of IR. Research on PI3K/Akt has advanced our understanding of the mechanisms underlying IR. Activation of this pathway exerts multiple actions, including Akt-mediated translocation of the glucose transporter GLUT4 to the cell membrane, thereby enhancing glucose uptake. In circumstances such as obesity and T2DM, the efficiency of insulin signaling through the PI3K/Akt pathway is generally diminished, resulting in reduced GLUT4 translocation and decreased glucose uptake by tissues (11). The gut microbiota and the endocannabinoid system (ECS), particularly through CB1 and CB2 receptors and PI3K signaling, engage in a bidirectional interaction that is fundamentally involved in controlling inflammation, maintaining gut barrier integrity, and modulating immune responses. Microbial composition influences ECS activity, while cannabinoid signaling adjusts the equilibrium between pro- and anti-inflammatory mediators, shaping systemic inflammatory outcomes. This dynamic crosstalk underscores the integrated role of microbiota and ECS pathways in maintaining immune homeostasis and identifies promising targets for the treatment of inflammatory and metabolic disorders (12). Although *B. thetaiotaomicron* has been shown to modulate glucose metabolism and inflammation (10), its role in molecular signaling pathways related to metabolic regulation, as well as its potential for managing T2DM as a commensal bacterium under investigation for its next-generation probiotic (NGP) potential, remains unclear and warrants further investigation with a focus on molecular insights and relevant metabolic parameters.

## 2. Objectives

This study aimed to assess the effects of *B. thetaiotaomicron* on metabolic parameters — including glycemic indices, IR markers, and lipid profiles — as well as on the expression of diabetes- and inflammation-related genes, and the abundance of targeted gut microbiota in a rat model of T2DM.

## 3. Methods

### 3.1. Materials

Materials were obtained as follows: *B. thetaiotaomicron* CCUG 10774 from the DSMZ (German Collection of Microorganisms and Cell Cultures) Institute; brain heart infusion agar (BHI) (CAS: 7558-79-4) from Merck Company (Darmstadt, Germany); streptozotocin (STZ) (CAS: 18883-66-4) and pentobarbital sodium salt (CAS: 57-33-0) from Sigma-Aldrich (St. Louis, USA); the standard diet from the Khorak Dam Pars Company (Tehran, Iran); casein from the Iran Caseinate Company (Karaj, Iran); and DL-methionine, vitamin, and mineral premix from Behroshd Company (Saveh, Iran). Assay kits for blood glucose and lipid profiles were from AUDIT, Delta Darman Part Co. (Tehran, Iran). Additional kits included a rat insulin ELISA kit (ZellBio GmbH, Lonsee, Germany); TRIzol reagent (Maxcell, Iran); Real time SYBR Green 2X master mix (Parstous, Mashhad, Iran); DNase I (RNase-free, 500 U; MO5401) and cDNA synthesis kit (RT5201) (Cinnagene Co., Tehran, Iran); and FavorPrepTM fecal DNA Isolation Mini Kit (Favorgen Co., Pingtung, Taiwan).

### 3.2. Preparation of Bacteroides thetaiotaomicron

*Bacteroides thetaiotaomicron* was cultured using BHI broth supplemented with hemin (5 μg/mL) and menadione (1 μg/mL) (Sigma-Aldrich, USA). Plates were incubated anaerobically at 37°C for 14 -18 hours using the Anoxomat™ MARK II system (80% N₂, 10% CO₂, and 10% H₂). Strain identity was confirmed using species-specific PCR targeting the 16S rRNA gene. Cultures were grown until reaching an optical density (OD₆₀₀) of 1, washed with sterile anaerobic phosphate-buffered saline (PBS), and set to a final concentration of 1×10⁹ CFU/mL for oral administration to rats (13).

### 3.3. Animal model and Treatments

Thirty-two male Wistar rats were sourced from the Pasteur Institute of Iran, weighing between 190 - 210 g and aged 8 weeks. They were housed under a 12:12-hour light–dark cycle (lights on from 06:00 to 18:00), at 22 ± 2°C, and 40 ± 6% humidity. Each cage housed 2 - 3 rats and was equipped with sterile hardwood chip bedding, with ad libitum access to a standard diet and autoclaved drinking water throughout the study. All experimental procedures were performed in accordance with the Iranian regulations concerning the use and care of experimental animals (14). Furthermore, all animal surgeries were approved by the Research Ethics Committee of Shahid Beheshti University of Medical Sciences (IR.SBMU.ENDOCRINE.REC.1402.042).

Following a one-week acclimation period, rats were randomly categorized into two main groups: A normal control (NC; n = 16) group fed a regular diet and another group fed a high-fat diet (HFD, n = 16) for 3 weeks. On the final day of week 3, after 12 hours of fasting, HFD rats received an intraperitoneal injection of STZ (30 mg/kg) dissolved in 0.1 M citrate buffer (pH = 4.5) to induce T2DM (15), while NC rats received citrate buffer only. T2DM was confirmed seven days later by fasting blood glucose (FBG) levels of 150 - 350 mg/dL. Subsequently, each main group (T2DM and NC) was randomly divided into two subgroups receiving either *B. thetaiotaomicron* in PBS or PBS alone via oral gavage for 5 weeks, resulting in four groups: (1) T2DM-PBS, (2) T2DM-B.t, (3) NC-PBS, and (4) NC-B.t. The sample size for each group was computed to be eight rats considering the equation (16, 17):


$$n=\frac{\left[(z_{1-\frac{\alpha }{2}}+\;z_{1-\beta }\;)\;²\;\times \;(s_{1}^{2}\;+\;s_{2}^{2})\right]}{\;d²\;}$$

n: Sample size per group

Z-(1-⍺/2): Z value corresponding to the selected confidence level (α is usually 0.05)

Z- (1- ᵦ): Z value corresponding to the statistical power (β is usually 0.2, power = 80%)

S₁²: Variance of the first group

S₂²: Variance of the second group: d: expected difference between the means of the two groups (effect size).

**Figure 1.**
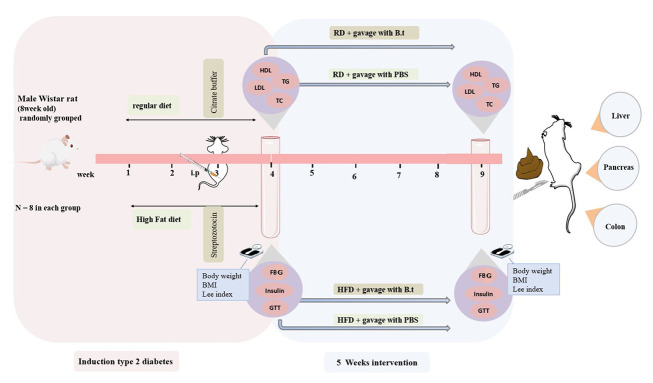
Schematic representation of the experimental design and timeline of the animal study

A schematic summary of the animal model and treatments is presented in Figure 1. 

### 3.4. Anthropometric Assessment

Anthropometric parameters including body weight (BW), naso-anal length, Body Mass Index (BMI) [weight (g)/length² (cm²)], and Lee Index [cube root of BW (g)/naso-anal length (cm)] were measured before and after the intervention, i.e., at weeks 4 and 9 (18).

### 3.5. Laboratory Analysis

For blood collection following a 12-hour fast or during the oral glucose tolerance test (OGTT), rats were anesthetized with sodium pentobarbital (45 mg/kg), and blood samples were obtained via a small tail tip incision.

#### 3.5.1. Fasting Blood Glucose and Lipid Measures

Biochemical analyses were conducted at weeks 4 and 9 (before and after the intervention). Measurements of FBG and lipid parameters including triglycerides (TG), total cholesterol (TC), high-density lipoprotein-cholesterol (HDL-C), and low-density lipoprotein-cholesterol (LDL-C) were performed using the Pictus 700 clinical chemistry analyzer, Diatron MI Plc (Budapest, Hungary). Serum fasting insulin levels were determined using a Sunrise ELISA reader (Tecan Co., Salzburg, Austria), and homeostatic model assessment of IR (HOMA-IR) was calculated considering the equation (19):


$$HOMA-IR\;=\frac{Fasting\;insulin\;(mU/L)\times FBG\;(mmol/L)}{22.5}$$

#### 3.5.2. Evaluation of Glucose Tolerance

Following a 12-hour fast, rats received an oral glucose solution (2 g/kg of 50% dextrose) and blood samples were collected at 15, 30, 60, 90, and 120 minutes post-administration (20).

### 3.6. Tissue Sampling

Rats were anesthetized via intraperitoneal injection of xylazine (5 mg/kg) and ketamine (90 mg/kg) (Bremer Pharma GmbH, Germany). Tissue samples from the pancreas, colon, and liver were taken; after rinsing in cold PBS, colon and liver were rapidly frozen in liquid nitrogen and kept at -80°C for molecular analyses.

#### 3.6.1. Histological Examination

Pancreatic tissue was immediately fixed in 10% neutral buffered formalin for histological analysis using hematoxylin and eosin (H&E). On histological examination, pancreatic islet architecture, islet size, cellular morphology, and degenerative changes were assessed.

#### 3.6.2. Determination of Target Genes in Liver and Colon Tissues

Total RNA was extracted from frozen liver and colon tissues using TRIzol reagent, followed by complementary DNA (cDNA) synthesis using a reverse transcription kit. Quantitative polymerase chain reaction (qPCR) was performed in duplicate using SYBR Green master mix on a StepOne System (Applied Biosystems by Life Technologies, Austin, TX, USA), with normalization to the elongation factor 2 (Eef2) gene. Primer sequences were listed in Appendix 1 in Supplementary File. Relative gene expression was determined using the 2^-ΔΔCt^ method. qPCR was performed with an initial denaturation at 95°C for 15 min, followed by 40 cycles of denaturation at 95°C for 15 s, primer-specific annealing for 20 s, and extension at 72°C for 25 s. A melt curve analysis was conducted to confirm amplification specificity.

### 3.7. Stool Sample Collection and DNA Extraction

Fresh stool samples were collected in sterile cups, immediately transferred on ice, and stored at -80°C. The FavorPrepTM stool DNA Isolation Mini Kit was used to extract genomic DNA from stool, according to the manufacturer's instructions. A NanoDrop™ spectrophotometer (Thermo Scientific, USA) was used to determine the concentration and purity of DNA. Extracted DNA was stored at -20°C.

#### 3.7.1. Analysis of Stool Microbiota

qPCR based on the SYBR Green method was performed using a Rotor Gene Q real time PCR system (QIAGEN, Germany) (21) to assess bacterial abundance in stool samples with 16S rRNA specific primers (Appendix 2 in Supplementary File). All reactions were run in duplicate. A universal primer amplification was carried out to confirm the presence of bacterial DNA. Target bacterial levels were established through a standard curve generated from serial dilutions of Escherichia coli, and the DNA copy number of each bacterium was derived by normalizing bacterial concentration to genome size.

### 3.8. Statistical analysis

Statistical differences between groups were analyzed using a two-way ANOVA for FBG, TG, TC, LDL-C, HDL-C, BW, Lee Index, BMI, insulin, and OGTT. Areas under the curve (AUC) were also calculated. Data normality was evaluated using the Shapiro-Wilk test before performing ANOVA. Group differences in gut microbiota abundance for each target bacterium and in the expression of diabetes-related and inflammation-related genes were analyzed using one-way ANOVA followed by the Bonferroni post hoc test. Results are presented as mean ± standard error of the mean (SEM), and P-value < 0.05 was considered statistically significant. All statistical analyses were performed using GraphPad Prism version 8.0 (GraphPad Software Inc., CA, USA).

## 4. Results

### 4.1. The Effect of Bacteroides thetaiotaomicron Administration on Anthropometric Indices

#### 4.1.1 Within Group Comparison Between Week 4 and 9

**Figure 2.**
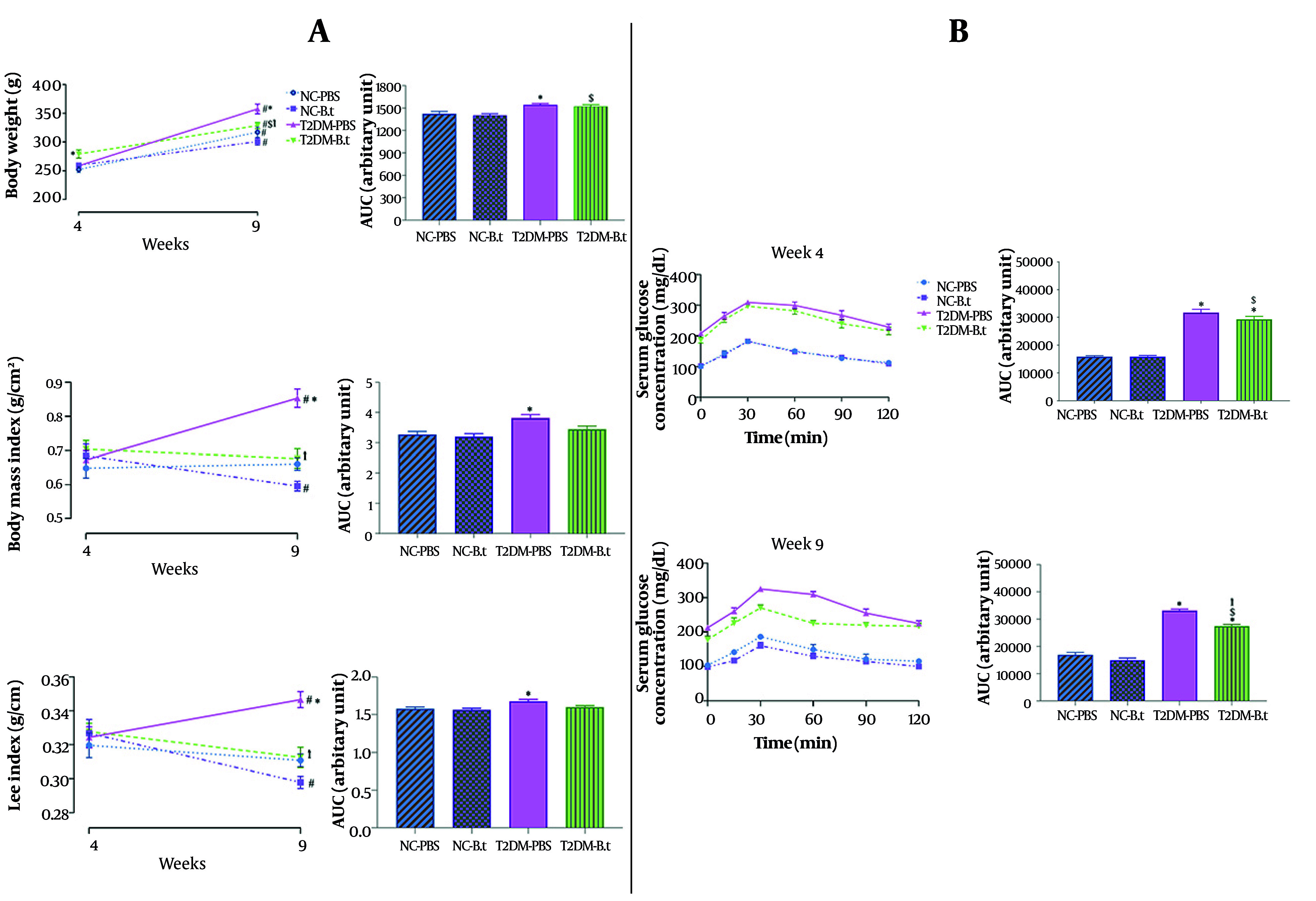
Within and between-groups comparison of anthropometric parameters and oral glucose tolerance test (OGTT) pre- and post-administration of *Bacteroides thetaiotaomicron* (B.t). A, anthropometric parameters [body weight (BW) and its area under the curve (AUC) body weight values, Body Mass Index (BMI) and its AUC BMI values, Lee Index and its AUC Lee Index values]; B, OGTT AUC glucose values in week 4 and week 9. Data are expressed as mean ± standard error of the mean (SEM) (n = 8); Statistically significant difference (P < 0.05) by post hoc Bonferroni’s two-way ANOVA is shown with different symbols as below: #: Within-group difference between week 4 and week 9; *: Compared to the NC-PBS group for their corresponding time points; †: Compared to the T2DM-PBS group for their corresponding time points; $: Compared to the T2DM-B.t group for their corresponding time points. Different groups: NC-PBS, normal control group received PBS; NC-B.t, normal control group received B.t; T2DM-PBS, type 2 diabetic group received PBS; T2DM-B.t, type 2 diabetic group received B.t; AUC, area under the curve.

Compared to week 4, BW at week 9 increased significantly by 26% and 16% in the NC-PBS and NC-B.t groups, respectively (both P < 0.0001). In the NC-B.t group, BMI and Lee Index decreased significantly by 13% and 9%, respectively (both P ≤ 0.03). As shown in Figure 2A, the T2DM-PBS group exhibited significant increases in BW (38%), BMI (27%), and Lee index (7%) (all P ≤ 0.011). In the T2DM-B.t group, only BW increased significantly (18%, P < 0.0001).

#### 4.1.2 Between Groups Comparison in Week 4 and 9

Type 2 diabetes mellitus-PBS compared to NC-PBS: Among those groups receiving PBS, no significant differences were observed between the T2DM-PBS and NC-PBS groups in week 4; after 5 weeks, the T2DM-PBS group exhibited significantly higher BW, BMI, and Lee index by 13%, 29%, and 11%, respectively (all P ≤ 0.0003).

Type 2 diabetes mellitus-B.t compared to T2DM-PBS: At week 9, the T2DM-B.t group had significantly lower BW, BMI, and Lee Index than the T2DM-PBS group by 8%, 21%, and 10%, respectively (all P ≤ 0.012). Although obesity indices in T2DM-B.t decreased over 5 weeks, they remained above those of the NC-PBS group (Figure 2A). 

#### 4.1.3 Between-Group Comparison Over the 5-Week Administration Period Assessed by Area Under the Curve

Over the 9-week period, the T2DM-PBS group showed significantly higher BW, BMI, and Lee Index than the NC-PBS group by 8%, 17%, and 6%, respectively (all P ≤ 0.030), as assessed by AUC (Figure 2A). 

### 4.2. The Effect of Bacteroides thetaiotaomicron Administration on Oral Glucose Tolerance Test

As shown in Figure 2B, the T2DM-PBS group had a higher glucose area under the curve (AUC) during the OGTT compared to the NC-PBS group at weeks 4 and 9, by 100% and 96%, respectively (both P < 0.0001). At week 9, the T2DM-B.t group had a 17% lower glucose AUC during the OGTT compared to the T2DM-PBS group (P < 0.0001); however, this intervention had no effect on OGTT in the NC-B.t group. In comparison to the NC-PBS group, the glucose AUC during the OGTT in the T2DM-B.t group was significantly higher in both weeks 4 and 9 by 85% and 62%, respectively (both P < 0.0001).

### 4.3. The Effect of Bacteroides thetaiotaomicron Administration on Metabolic Parameters

#### 4.3.1 Within-Group Comparison Between Weeks 4 and 9

**Figure 3.**
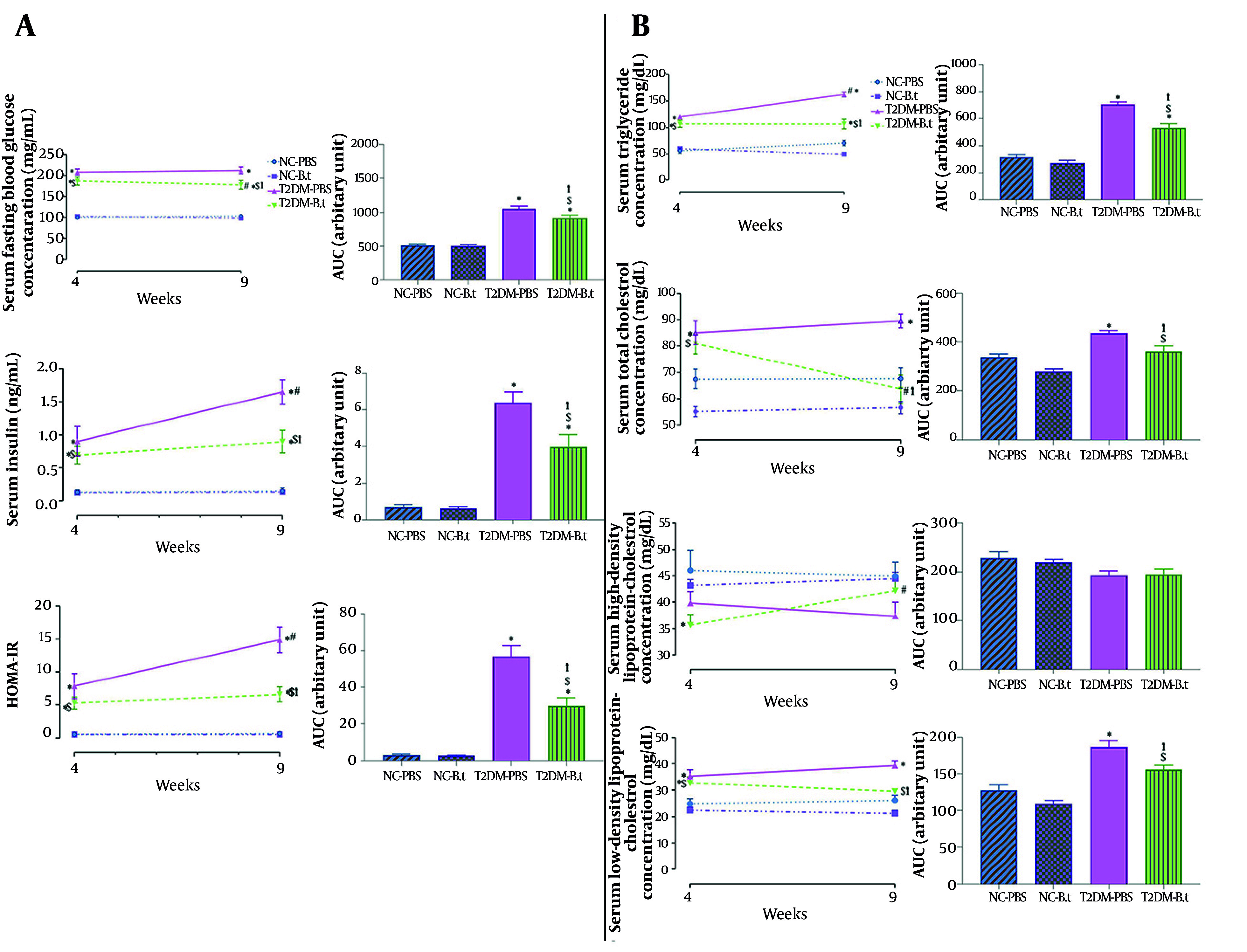
Within and between-groups comparison of fasting blood glucose (FBG), insulin, homeostatic model assessment of insulin resistance (HOMA-IR), and lipid profile pre- and post-administration of *Bacteroides thetaiotaomicron* (B.t). A, FBG and its AUC FBG values, insulin and its AUC insulin values, HOMA-IR and its AUC HOMA-IR values; B, lipid profile [triglycerides (TG) and its AUC TG values, total cholesterol (TC) and its AUC TC values, high-density lipoprotein-cholesterol (HDL-C) and its AUC HDL-C values, low-density lipoprotein-cholesterol (LDL-C) and AUC LDL-C values]. Data are expressed as mean ± SEM (n = 8); Statistically significant difference (P < 0.05) by post hoc Bonferroni’s two-way ANOVA is shown with different symbols as below: #: Within-group difference between week 4 and week 9; *: Compared to the NC-PBS group for their corresponding time points; †: Compared to the T2DM-PBS group for their corresponding time points; $: Compared to the T2DM-B.t group for their corresponding time points. Different groups: NC-PBS, normal control group received PBS; NC-B.t, normal control group received B.t; T2DM-PBS, type 2 diabetic group received PBS; T2DM-B.t, type 2 diabetic group received B.t; AUC, area under the curve.

Figure 3A showed the comparison of levels of FBG, insulin, HOMA-IR, and different lipid parameters in week 9 compared to week 4 in each group. The T2DM-B.t group had significantly lower FBG and TC by 5% and 21%, respectively (both P ≤ 0.024), and significantly higher HDL-C by 18% (P = 0.029) at week 9. The T2DM-PBS group had significantly higher insulin, HOMA-IR, and TG by 83%, 89%, and 36%, respectively (all P ≤ 0.002). Low-density lipoprotein-cholesterol showed no significant changes in any group.

#### 4.3.2 Between-Groups Comparison in Weeks 4 and 9

Type 2 diabetes mellitus-PBS compared to NC-PBS: Comparisons between the T2DM-PBS and NC-PBS groups showed that at both weeks 4 and 9, the T2DM-PBS group had higher FBG (108% and 105%), insulin (569% and 966%), HOMA-IR (1283% and 2115%), TG (114% and 131%), TC (26% and 32%), and LDL-C (42% and 50%) (all P ≤ 0.009). HDL-C levels were not significantly lower in the T2DM-PBS group by 14% and 17% at weeks 4 and 9, respectively (both P ≥ 0.182) (Figure 3A and B).

Type 2 diabetes mellitus-B.t compared to T2DM-PBS: As expected in week 4, there were no significant differences in glucose and lipid parameters between the two diabetic groups. In week 9, the T2DM-B.t group had lower levels of FBG, insulin, HOMA-IR, TG, TC, and LDL-C by 16%, 46%, 56%, 34%, 29%, and 25%, respectively (all P ≤ 0.006); whereas HDL-C was 13% higher but not statistically significant (P = 0.971). Although administration of *B. thetaiotaomicron* for 5 weeks was accompanied by significantly lower levels of these biochemical parameters (except HDL-C), their blood levels remained elevated compared to those in the NC-PBS group (Figure 3A and B).

#### 4.3.3 Between-Groups Comparison Over the 5-Week Treatment Period Assessed by Area Under the Curve

Type 2 diabetes mellitus-PBS compared to NC-PBS: After 5 weeks, compared to the NC-PBS group, the T2DM-PBS group showed significantly higher levels of FBG, insulin, HOMA-IR, TG, TC, and LDL-C by 107%, 781%, 1734%, 123%, 29%, and 46%, respectively (all P ≤ 0.0004).

Type 2 diabetes mellitus-B.t compared to T2DM-PBS: Administration of *B. thetaiotaomicron* in the T2DM-B.t group was associated with significantly lower levels of FBG, insulin, HOMA-IR, TG, TC, and LDL-C by 13%, 38%, 48%, 24%, 17%, and 17% (all P ≤ 0.044). Despite these reductions, their blood levels remained significantly higher than in the NC-PBS group.

NC-B.t compared to NC-PBS: After 5 weeks of administration of *B. thetaiotaomicron*, no significant differences were observed in blood glucose or lipid parameters between the NC-B.t and NC-PBS groups (Figure 3A and B).

### 4.4. The Effect of Bacteroides thetaiotaomicron on the Expression of Diabetes-Related Genes in the Liver

**Figure 4.**
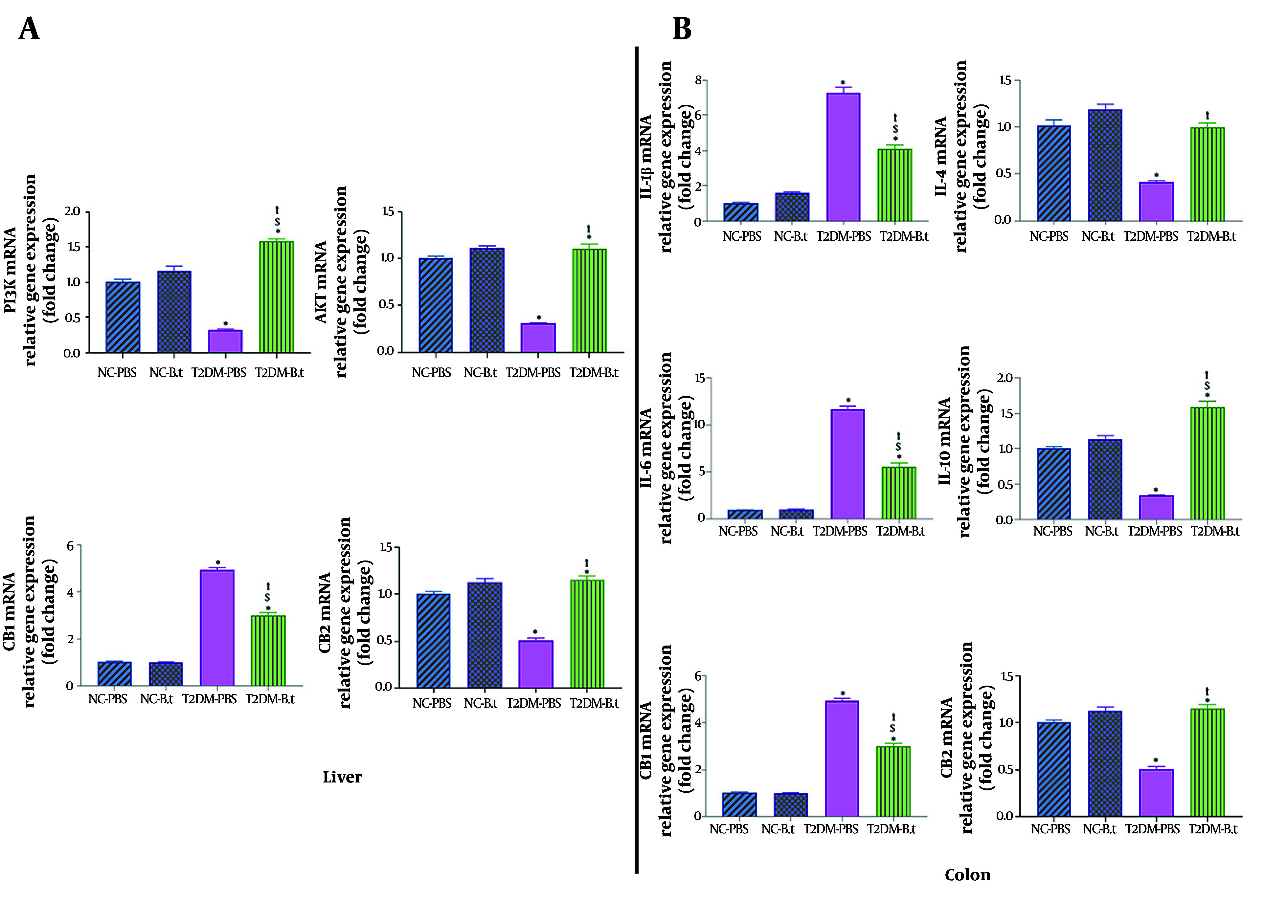
Effect of *Bacteroides thetaiotaomicron* (B.t) on the expression of genes in the liver and the colon. A) PI3K, Akt, CB1, and CB2 in the liver; B) IL-1β, IL-4, IL-6, IL-10, CB1, and CB2 in the colon. Data are expressed as mean ± standard error of the mean (SEM) (n = 8); Statistically significant difference (P < 0.05) by post hoc Bonferroni’s one-way ANOVA is shown with different symbols as below: *: Compared to the NC-PBS group for their corresponding time points; †: Compared to the T2DM-PBS group for their corresponding time points; $: Compared to the T2DM-B.t group for their corresponding time points. Different groups: NC-PBS: normal control group received PBS; NC-B.t, normal control group received B.t; T2DM-PBS, type 2 diabetic group received PBS; T2DM-B.t, type 2 diabetic group received B.t; PI3K, phosphoinositide 3-kinase; Akt, protein kinase B; CB1, cannabinoid receptor 1; CB2, cannabinoid receptor 2; IL-1β, interleukin-1β; IL-4, interleukin-4; IL-6, interleukin-6; IL-10, interleukin-10.

To investigate the role of *B. thetaiotaomicron* in signaling pathways, the expression of genes related to glucose metabolism was analyzed in the liver. The expression levels of CB1, CB2, PI3K, and Akt were evaluated. CB1 was significantly up-regulated (6.54-fold; P < 0.0001) in the T2DM-PBS group versus the NC-PBS group. In contrast, PI3K, Akt, and CB2 were significantly down-regulated (3.14-, 3.24-, and 1.96-fold, respectively; all P < 0.0001). Following *B. thetaiotaomicron* supplementation, CB1 was reduced (1.81-fold; P < 0.0001), whereas PI3K, Akt, and CB2 expressions were significantly increased (4.91-, 3.55-, and 2.26-fold, respectively; all P < 0.0001) in the T2DM-B.t group compared to the T2DM-PBS group (Figure 4A).

### 4.5. The Effect of Bacteroides thetaiotaomicron on the Expression of Inflammation-Related Genes in the Colon

As shown in Figure 4B, the T2DM-PBS group had significantly higher IL-1β, IL-6, and CB1 expressions (7.20-, 11.66-, and 4.92-fold, respectively; all P < 0.0001) and lower IL-4, IL-10, and CB2 levels (2.47-, 2.89-, and 1.86-fold, respectively; all P ≤ 0.035) compared to NC-PBS. After intervention, the expression levels of IL-1β, IL-6, and CB1 were significantly down-regulated in the T2DM-B.t group (1.76-, 2.10-, and 1.64-fold, respectively; all P < 0.0001). Conversely, the expressions of IL-4, IL-10, and CB2 were significantly up-regulated (2.43-, 4.6-, and 1.47-fold, respectively; all P ≤ 0.0009) in comparison to the T2DM-PBS group. However, *B. thetaiotaomicron* administration to the NC-B.t group was not associated with significant changes.

### 4.6. The Effect of Bacteroides thetaiotaomicron on Gut Microbiota Composition

**Figure 5.**
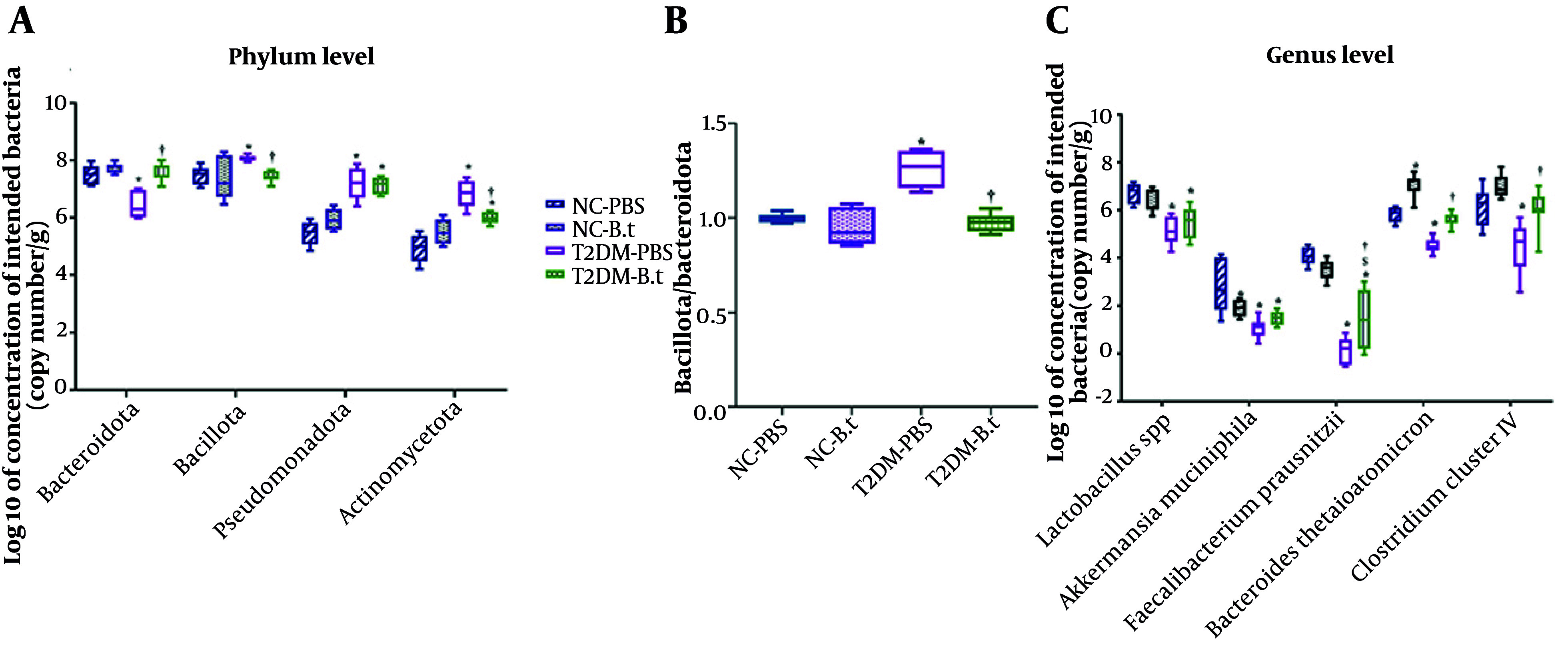
Effect of *Bacteroides thetaiotaomicron* (B.t) on the targeted gut microbial composition in the different study groups. A, gut microbiome composition at the phylum levels; B, ratio of Bacillota/Bacteroidota; C, gut microbiome composition at the genus and species levels. The gut microbial composition was obtained through 16S rRNA; Data are expressed as mean ± SEM (n = 8); statistically significant difference (P < 0.05) by post hoc Bonferroni’s one-way ANOVA is shown with different symbols as below. *: Compared to the NC-PBS group for their corresponding time points; †: Compared to the T2DM-PBS group for their corresponding time points; $: Compared to the T2DM-B.t group for their corresponding time points. Different groups: NC-PBS, normal control group received PBS; NC-B.t, normal control group received B.t; T2DM-PBS, type 2 diabetic group received PBS; T2DM-B.t, type 2 diabetic group received B.t.

As shown in Figure 5A, at the phylum level, the T2DM-PBS group had significantly higher copy numbers of Actinomycetota (38%, P < 0.0001), Pseudomonadota (32%, P < 0.0001), and Bacillota (8%, P = 0.030) compared with the NC-PBS group, while Bacteroidota copy number was significantly lower (14%, P < 0.0001). Administration of *B. thetaiotaomicron* to the T2DM-B.t group reduced Actinomycetota and Bacillota copy numbers by 12% (P = 0.001) and 8% (P = 0.020), while increasing Bacteroidota by 19% (P < 0.0001), compared to the T2DM-PBS group. However, no significant changes in the copy numbers of any of these phyla were observed in the NC-B.t group.

As shown in Figure 5B, in comparison to the NC-PBS group, the T2DM-PBS group exhibited a significantly higher Bacillota/Bacteroidota ratio (26%, P < 0.0001). However, the Bacillota/Bacteroidota ratio in the T2DM-B.t group compared to the T2DM-PBS group was significantly decreased (23%, P < 0.0001).

At the genus level (Figure 5C), the T2DM-PBS group had significantly lower copy numbers of *Lactobacillus* spp. (24%), *A. muciniphila* (63%), *F. prausnitzii* (97%), *B. thetaiotaomicron* (23%), and *Clostridium* cluster IV (28%) (all P ≤ 0.001), compared to the NC-PBS group. *B. thetaiotaomicron* administration to the T2DM-B.t group increased *F. prausnitzii* (1043%, P=0.009), *B. thetaiotaomicron* (24%, P<0.0001), and *Clostridium* cluster IV (35%, P = 0.003), compared to the T2DM-PBS group. However, although the copy numbers of A. muciniphila and Lactobacillus spp. increased by 41% and 7%, respectively, these changes were not statistically significant.

### 4.7. Histological Examination

**Figure 6.**
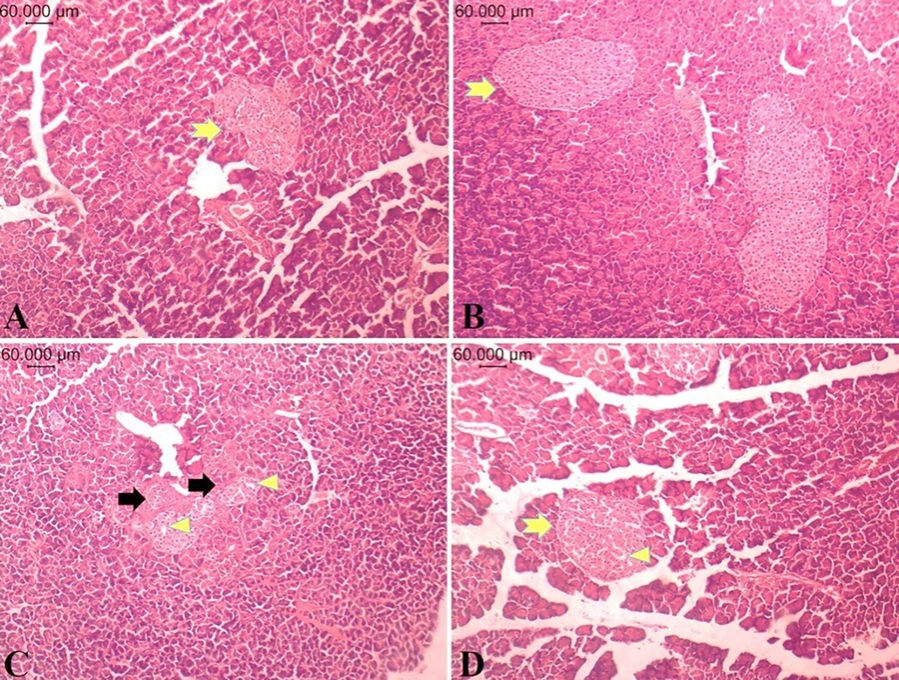
Histopathological findings of the pancreatic tissues in the different study groups. A, NC-PBS; B, NC-B.t; C, T2DM-PBS; and D, T2DM-B.t. Pancreatic specimens were prepared and examined under a light microscope (Olympus SZX16, Japan), H&E stain; 10X magnification; Normal islets (yellow arrow), islet atrophy (black arrow), destruction and vacuolation (arrowhead). Different groups: NC-PBS, normal control group received PBS; NC-B.t, normal control group received B.t; T2DM-PBS, type 2 diabetic group received PBS; T2DM-B.t, type 2 diabetic group received B.t.

Histological examination showed normal pancreatic islet structure and cytomorphology in the NC-PBS and NC-B.t groups (Figure 6A and B). In the T2DM-PBS group, atrophic pancreatic islets with a reduction in overall size and degeneration and vacuolization of islet cells were observed (Figure 6C). In the T2DM-B.t group, the structure and organization of pancreatic islets were comparable with those of the T2DM-PBS group; furthermore, the severity of cellular changes was significantly reduced (Figure 6D). 

## 5. Discussion

This experimental study investigated the impact of *B. thetaiotaomicron* on metabolic parameters, diabetes- and inflammation-related gene expression, and gut microbiota composition in a T2DM animal model. The findings demonstrated that administration of *B. thetaiotaomicron* was associated with reductions in anthropometric measures, improvements in glycemic indices and IR markers, as well as decreased levels of all serum lipids except for HDL-C. Furthermore, following this intervention, the expression of PI3K, Akt, CB1, and CB2 genes was regulated in the liver. Additionally, the administration of *B. thetaiotaomicron* significantly down-regulated the expression of pro-inflammatory genes IL-1β, IL-6, and CB1 in the colon, while up-regulating expression of anti-inflammatory genes IL-4, IL-10, and CB2. Moreover, significant changes in targeted gut microbial composition were detected at both phylum and genus levels, i.e., a reduction in the abundance of Bacillota and Actinomycetota and an increase in the Bacteroidota phylum. Following treatment with *B. thetaiotaomicron*, the Bacillota/Bacteroidota ratio in the T2DM group was significantly reversed. At the genus level, increases in *F. prausnitzii*, *B. thetaiotaomicron*, and *Clostridium* cluster IV were observed. However, despite the increase in *A. muciniphila *and *Lactobacillus* spp. copy numbers, these changes were not statistically significant.

Among NGPs, the impact of bacteria such as *A. muciniphila* on diabetes has been extensively investigated, demonstrating a potential to enhance glucose metabolic regulation and reduce IR (22). According to clinical data, an increase in the abundance of Bacteroides enhances glucose metabolism (23), making these bacteria a promising new approach to microbiota-driven T2DM management. Bacteria of the *Bacteroidota phylum* constitute a substantial portion of the healthy gut microbiota, with *B. thetaiotaomicron* serving as a prominent and well-characterized representative species. This Gram-negative obligate anaerobic bacterium thrives in symbiosis with its host, and has recently been recognized as a promising candidate among NGP. In the human gut, *B. thetaiotaomicron* supports survival of the microbiota and host health by degrading and metabolizing complex glycans, such as intestinal mucus (24). A wide range of disorders have been linked to shifts in the intestinal population of beneficial bacteria, including *B. thetaiotaomicron* (9, 25). Previous studies have investigated the role of *B. thetaiotaomicron* in modulating inflammation- and mucus-related gene expression in various diseases, excluding T2DM (9, 26). As reported by Lee et al., this bacterium reduces inflammation associated with IR by improving intestinal barrier function, increasing insulin sensitivity, and improving glucose metabolism (27). Furthermore, *B. thetaiotaomicron* modulates bile acid metabolism through its interaction with nuclear receptors such as the farnesoid X receptor, a key regulator of glucose and lipid homeostasis (28).

According to our findings, five weeks of *B. thetaiotaomicron* treatment improved obesity-related indices in the T2DM-B.t group compared to the T2DM-PBS group. In line with our findings, Suastika et al. reported significant reductions in BW in diabetic rats using other probiotics such as *Lactobacillus rhamnosus* (29). This outcome is promising, as preventing weight gain and achieving modest weight reduction can improve glucose metabolism and reduce diabetes-related complications in patients with T2DM (29, 30).

In the present study, administration of *B. thetaiotaomicron* led to significant improvements in glycemic parameters in the T2DM-B.t group compared to the T2DM-PBS group. Consistent with our findings, previous studies have demonstrated that probiotic strains such as *Lactobacillus* Q14 and G15 and *L. paracasei* NL41 positively influence metabolic parameters associated with T2DM, including improvements in FBG, IR, and OGTT, thereby highlighting their potential role in the management of T2DM (31, 32).

In the current study, lower TC and TG levels in the T2DM-B.t group suggest a potential improvement in dyslipidemia associated with T2DM following *B. thetaiotaomicron* administration. These effects may be mediated by increased production of short-chain fatty acids (SCFAs), which are known to arise from the fermentation of dietary fibers by beneficial gut bacteria like *B. thetaiotaomicron* (33). A recent study conducted in an animal model of non-alcoholic fatty liver disease (NAFLD) showed that treatment with *B. thetaiotaomicron* in mice significantly improved hyperlipidemia, an important metabolic abnormality of NAFLD, by improving lipid metabolism through SCFAs (34). Another study reported that treatment with *B. thetaiotaomicron* in obese mice was associated with weight loss, improved OGTT, and enhanced lipid profiles, reflecting its role as a modulator of metabolic processes related to glucose homeostasis (27). In contrast to the aforementioned studies, Cho et al. reported that *B. thetaiotaomicron* exacerbated metabolic disorders by increasing lipid digestion and absorption in high-fat diet (HFD)-fed mice, leading to weight gain and impaired glucose tolerance. They attributed this effect to the ability of *B. thetaiotaomicron* to regulate fatty acid transporters and suppress ANGPTL4, an inhibitor of lipoprotein lipase (35). These inconsistencies may be related to differences in experimental conditions such as variations in the composition of the mouse gut microbiota or slight differences in diet formulation, leading to variability in the observed metabolic outcomes. Additionally, interactions between *B. thetaiotaomicron* and other microbial species may vary, further influencing the metabolic response.

The role of *B. thetaiotaomicron* in metabolism is multifaceted, encompassing both direct involvement in metabolic processes and modulation of host gene expression. These functions are fundamental for the host-bacterial interaction, enabling the host to partially regulate gut microbiota composition and enhance its metabolic homeostasis (36). This study showed that *B. thetaiotaomicron* intervention regulated the expression of diabetes-related genes, including PI3K, Akt, CB1, and CB2 in the liver. Consistent with our results, a study on T2DM reported that probiotic *L. plantarum* HAC01 reduced endogenous glucose production in the liver, which may be accompanied by activation of the butyric acid-AMPK and PI3K/Akt pathways (37). Another study supports our results by demonstrating that *L. paracasei* HII01 enhanced insulin sensitivity through the restoration of Akt activation, a key component of the PI3K/Akt pathway (38).

The ECS, including CB1 and CB2 receptors, is a lipid signaling network that regulates numerous biochemical processes; it plays a crucial role in the microbiota–gut-brain axis and is important for regulating inflammation, modulating inflammatory responses, and influencing various physiological states in the body (39). Preclinical studies increasingly support the idea that targeting the ECS may yield beneficial effects on T2DM, positioning this complex lipid signaling network as a potential source for novel treatment strategies for T2DM (40, 41). The CB1 receptor is implicated in the regulation of energy balance and glucose metabolism. It can modulate insulin signaling pathways, especially the PI3K/Akt pathway. Insulin binding to its receptor leads to the phosphorylation of insulin receptor substrate 1 (IRS1), which in turn activates PI3K. PI3K converts PIP2 to PIP3, leading to Akt activation. Activated Akt orchestrates several downstream effects, including the translocation of GLUT4 to the plasma membrane (enhancing glucose uptake) and promotion of glycogen synthesis. One of the targets of Akt is the mammalian target of rapamycin (mTOR), a central regulator of cell growth, protein synthesis, and lipid metabolism. Dysregulation of these pathways, such as chronic CB1 activation, may impair IRS1 phosphorylation, disrupt PI3K/Akt/mTOR signaling, and contribute to IR, increased adipogenesis, and the development of T2DM (42).

Activation of CB2 receptors in immune cells, on the other hand, leads to a decrease in the activation of the transcription factor NF-κB, which directly suppresses the expression of inflammatory cytokines, such as IL-6, IL-1β, and TNF-α. The reduction of IL-6 secretion in inflammatory and metabolic processes not only contributes to better control of systemic inflammation but can also improve IR and metabolic complications caused by T2DM (43). Thus, modulating gene expression within this pathway may offer a therapeutic strategy to improve glucose homeostasis (44).

Our study also examined the expression of inflammation-related genes in the liver and colon. The observed down-regulation of expression of genes related to pro-inflammatory cytokines in the T2DM-B.t group suggests an anti-inflammatory role of *B. thetaiotaomicron*, and its potential involvement in reducing chronic low-grade inflammation and IR in T2DM (43).

Consistent with our findings, previous studies have demonstrated that the expression of IL-1β and IL-6 genes increased in diabetic patients (45), while the expression of IL-4 and IL-10 genes decreased (46). Although the anti-inflammatory effects of *B. thetaiotaomicron* in T2DM were not addressed intensively, this issue was examined in other diseases. A recent study reported the anti-inflammatory effect of *B. thetaiotaomicron* in a mouse model of dextran sodium sulfate (DSS)-induced colitis, as indicated by decreased levels of inflammatory factors, particularly IL-6 (25). In a separate study, the effects of *B. thetaiotaomicron *administration on colitis in DSS and IL-10 knockout models of inflammatory bowel disease showed that *B. thetaiotaomicron* modulates intestinal inflammation (9). Pang et al. investigated the therapeutic potential of *B. thetaiotaomicron *in a murine model of allergic airway inflammation and reported an increase in IL-10-expressing regulatory T cells (Tregs). The authors suggested that *B. thetaiotaomicron* alleviated allergic airway disease through the promotion of IL-10-mediated immune regulation and activation of Tregs (47). In another study, oral administration of *B. thetaiotaomicron* in mice was associated with enhanced anti-inflammatory responses, characterized by suppression of pro-inflammatory cytokines and concomitant up-regulation of the anti-inflammatory cytokines. This immunomodulatory effect was linked to elevated expression of toll-like receptor 9 (TLR9) and chitinase-like protein 1 activation (48). Consistent with these reports, our study demonstrated that *B. thetaiotaomicron *administration could reduce the expression of diabetes- and inflammation-related genes in rats.

In this study, to investigate the effect of *B. thetaiotaomicron *administration on the modification of the gut microbiota, various targeted bacterial taxa were analyzed, including Actinomycetota, Bacillota, Bacteroidota, and Pseudomonadota (at the phylum level) and *Lactobacillus* spp., *A. muciniphila*, *F. prausnitzii*, *B. thetaiotaomicron*, and *Clostridium* cluster IV (at the genus level). These taxa are known to play crucial roles in modulating inflammation, IR and sensitivity, glucose tolerance, the integrity of the intestinal barrier, and endotoxin translocation through different pathways. Fermentation of nutrients by these genera produces metabolites such as short-chain fatty acids (SCFAs) (including butyrate, propionate, and acetate), branched-chain amino acids, indoles, imidazole, and succinates (49-51).

In our study, *B. thetaiotaomicron* administration was associated with a decrease in the copy numbers of Bacillota and Actinomycetota and an increase in Bacteroidota, as well as a reversal of the Bacillota/Bacteroidota ratio. The genera *F. prausnitzii* and *Clostridium* cluster IV increased significantly, whereas the increases in *A. muciniphila* and Lactobacillus spp. did not reach statistical significance. Consistent with our findings, a previous study on the probiotic *L. plantarum* HAC01 showed that this probiotic could modulate gut microbiota to improve metabolic health; it increased the abundance of beneficial bacteria like *A. muciniphila*, while reducing pathobiont bacteria such as Pseudomonadota, thereby supporting their therapeutic potential in managing metabolic disorders by promoting gut health and modulating metabolic pathways (37). Similarly, in a study on HFD-fed mice, a reduction in the Bacillota/Bacteroidota ratio following *B. thetaiotaomicron* intervention was observed (34). These consistent findings support the notion that a decreased Bacillota/Bacteroidota ratio reflects attenuation of disease-associated dysbiosis. Zocco et al. reported that modulation of gut microbiota by *B. thetaiotaomicron* may restore microbial balance and enhance metabolic health (36).

In addition to cytokine-mediated modulation, the gut microbiota plays a critical role in host metabolism and immune homeostasis. Dysbiosis, defined as an imbalance in microbial composition, can disrupt gut barrier integrity and promote systemic inflammation. This occurs partly through increased gut permeability, allowing bacterial components like lipopolysaccharides (LPS) (52) to translocate and activate TLR4-dependent immune responses and promote IR and β-cell dysfunction. Increased gut permeability also exacerbates metabolic endotoxemia. Moreover, the gut microbiota modulates bile acid metabolism and the ECS, thereby further impacting IR and inflammation. Thus, therapeutic strategies aimed at modulating the gut microbiota with probiotics by targeting the ECS represent promising avenues for the management of T2DM (53).

Islet dysfunction is well established in T2DM, although the underlying causal factors remain unclear. A reduction in β-cell mass, as demonstrated in diabetic models, has been proposed as a contributing factor (54). In our study, administration of *B. thetaiotaomicron* to the T2DM-B.t group attenuated the severity of morphological alterations in pancreatic tissue, while the structure and organization of islet cells tended toward normal features. In line with these findings, Zhao et al. reported the positive effects of Bifidobacterium longum on diabetes-induced pancreatic tissue damage in animal models (55).

In summary, this study explored the therapeutic potential of *B. thetaiotaomicron* to improve metabolic dysregulation, inflammation, and gut microbiota imbalance in a well-established T2DM rat model that closely mimics human disease. Supplementation with *B. thetaiotaomicron* improved BW, glucose metabolism, lipid profiles, and restored microbial composition, along with modulating the expression of diabetes- and inflammation-related genes. A major strength of this study was the comprehensive evaluation of interconnected metabolic, molecular, microbial, and histological parameters, including anthropometric indices, glucose and lipid metabolism, inflammatory markers, and gut microbiota composition. In addition to assessing genes involved in PI3K/Akt and inflammatory pathways, ECS-related genes in both the liver and colon were also evaluated. While these findings provide valuable insights and offer promising implications for potential translation to human health, the study was limited by its focus on a single bacterial strain, and further clinical investigations are warranted to confirm applicability.

### 5.1. Conclusions

This experimental study demonstrated that *B. thetaiotaomicron* administration improves anthropometric measures, glycemic indices, IR, and lipid profiles, and regulates the expression of diabetes- and inflammation-related genes, alongside modification of gut microbiota composition in a T2DM rat model. This study suggests that *B. thetaiotaomicron* may hold potential NGP candidate with relevance to glucose regulation and T2DM. Future research should investigate the synergistic effects of *B. thetaiotaomicron* in combination with other probiotics, alongside comprehensive safety assessments and human clinical trials.

## supplementary material

ijem-23-4-168629.pdf

## Data Availability

All the data that support the findings of this study are available from the corresponding author, upon reasonable request.
